# Behavioral Fever Drives Epigenetic Modulation of the Immune Response in Fish

**DOI:** 10.3389/fimmu.2018.01241

**Published:** 2018-06-04

**Authors:** Sebastian Boltana, Andrea Aguilar, Nataly Sanhueza, Andrea Donoso, Luis Mercado, Monica Imarai, Simon Mackenzie

**Affiliations:** ^1^Interdisciplinary Center for Aquaculture Research (INCAR), Department of Oceanography, Biotechnology Center, University of Concepción, Concepción, Chile; ^2^Grupo de Marcadores Inmunológicos, Facultad de Ciencias, Instituto de Biología, Pontificia Universidad Católica de Valparaíso, Valparaíso, Chile; ^3^Laboratory of Immunology, Center of Aquatic Biotechnology, Department of Biology, Faculty of Chemistry and Biology, University of Santiago of Chile, Santiago, Chile; ^4^Institute of Aquaculture, University of Stirling, Stirling, United Kingdom

**Keywords:** behavioral fever, gene regulation, lymphocyte proliferation, cytokine release, epigenetic modification

## Abstract

Ectotherms choose the best thermal conditions to mount a successful immune response, a phenomenon known as behavioral fever. The cumulative evidence suggests that behavioral fever impacts positively upon lymphocyte proliferation, inflammatory cytokine expression, and other immune functions. In this study, we have explored how thermal choice during infection impacts upon underpinning molecular processes and how temperature increase is coupled to the immune response. Our results show that behavioral fever results in a widespread, plastic imprint on gene regulation, and lymphocyte proliferation. We further explored the possible contribution of histone modification and identified global associations between temperature and histone changes that suggest epigenetic remodeling as a result of behavioral fever. Together, these results highlight the critical importance of thermal choice in mobile ectotherms, particularly in response to an infection, and demonstrate the key role of epigenetic modification to orchestrate the thermocoupling of the immune response during behavioral fever.

## Highlights

- Behavioral fever promotes the coordinate of lymphocyte proliferation and proinflammatory cytokine release.
- Temperature primes and activates a specific subset-of mRNA transcripts relevant to the innate and adaptive immune response.
- Behavioral fever modifies the histone and transcriptional landscape.
- Behavioral fever is an integrative signal potentiating the immune response.

## Introduction

Thermoregulatory behavior is a critical factor influencing the functional responses of individuals in aquatic environments (1). Surprisingly, it remains unknown how ectothermic vertebrates regulate thermal preference, where central thermostats are located in the brain and how variations in thermal choice impact upon molecular and cellular interactions (2–4). Behavioral fever has been shown to impact the immune response by drive specific modification of the molecular regulation (5, 6). However, this does not involve that fever has an adaptive impact, but that it can have a key incidence on the survival depending on the specific pathological conditions (7) where a balance between the killing of invading pathogens and the specific molecular/cellular response of each tissue have to be taken into account (8). For example, it has recently been shown that some viruses carry genes that specifically inhibit the fever response to increase their growth (9, 10), or as in the case of *Neisseria meningitidis*, the increase of the temperature leads to expression of mechanisms to specifically avoid the host immune responses that are triggered during fever (11).

In general, behavioral fever response has been described across a range of fish species highlighting the ubiquitous nature of this response. Acute variations in body temperature achieved by displacement through a thermal gradient may influence the regulation of the immune response at several levels of interaction reaching from molecular to whole organ response. In environmental settings where thermal choice behavior is limited by the environment such as extreme temperatures or constant thermal conditions acute stress responses and immune compromise are observed (5). In thermally stressed fish, for example, increased temperatures with no behavioral choice the thermal stress negatively impacts upon the glucocorticoid response (GC), innate immunity (12, 13), the oxidative stress response (14, 15), and causes reduced lymphocyte numbers and proliferation rates (16). Interestingly, under adequate environmental conditions that include a thermal choice fish are able to successfully orchestrate regulatory responses to potentiate immunity (17) or improve metabolic and growth performance (18). Behavioral fever in response to pathogen challenge has been shown in fish as a response to bacterial (tilapia), cytokine (trout), and viral pathogens (carp, zebrafish), in lizards as a response to lipopolysaccharide (LPS) of bacterial wall of *Escherichia coli* (19) and invertebrates highlighting the evolutionary importance of this response in ectotherms (20–23).

Existing evidence indicates that behavioral fever is an evolutionarily conserved response with significant adaptive value (9, 24–28), although there is a lack of scientific knowledge regarding the underpinning mechanisms influenced by thermal coupling of the immune response during behavioral fever. The mechanisms by which increased temperature affect the defense response are complex and may include epigenetic changes impacting specific immunological processes. Recent reports have documented the role of the epigenetic regulation on the immunological pathways underlying the defense response (29, 30). For example, mammal macrophages previously challenged with fungal cell-wall compound β-glucan or bacterial LPS exhibit genome-wide changes in the trimethylation of histone H3 at Lys4 (H3K4me3), monomethylation of histone H3 at Lys4 (H3K4me1), and acetylation of histone H3 at Lys27 (H3K27ac), as well as transcriptional remodulation (31). In particular, the molecular mechanisms affected or influenced by temperature choice and the translation into potentiated immune response remain unknown. In ectotherms, the influence of thermoregulation upon gene expression has been supported by correlations between temperature, regulatory response, and variation in gene expression (32, 33). However, the contribution of epigenetic regulation including DNA methylation and histone modification remains unexplored [e.g., Streelman et al. (34); Baalsrud et al. (35); and Mallard et al. (36)].

In this study, we proposed to explore the epigenetic regulatory mechanisms influenced by behavioral fever. To test this hypothesis, we used *Atlantic salmon, Salmo salar*, that had access either to a thermal choice through a thermal gradient or were held at a constant temperature. Experimental animals were subjected to a viral challenge, infectious pancreatic necrosis virus (IPNv). Using this dynamic experimental set up, we are able to test for an association between gene regulation, epigenetic modification, cellular response, and survival to define the impact of temperature on the response to viral infection. Our findings highlight the role of the epigenetic modifications driven by behavioral fever that contribute to the observed variation in gene expression, the variation in lymphocyte cell populations and cytokine production that ultimately impact on survival.

## Materials and Methods

### Animal and Experimental Conditions

**Figure 1:**
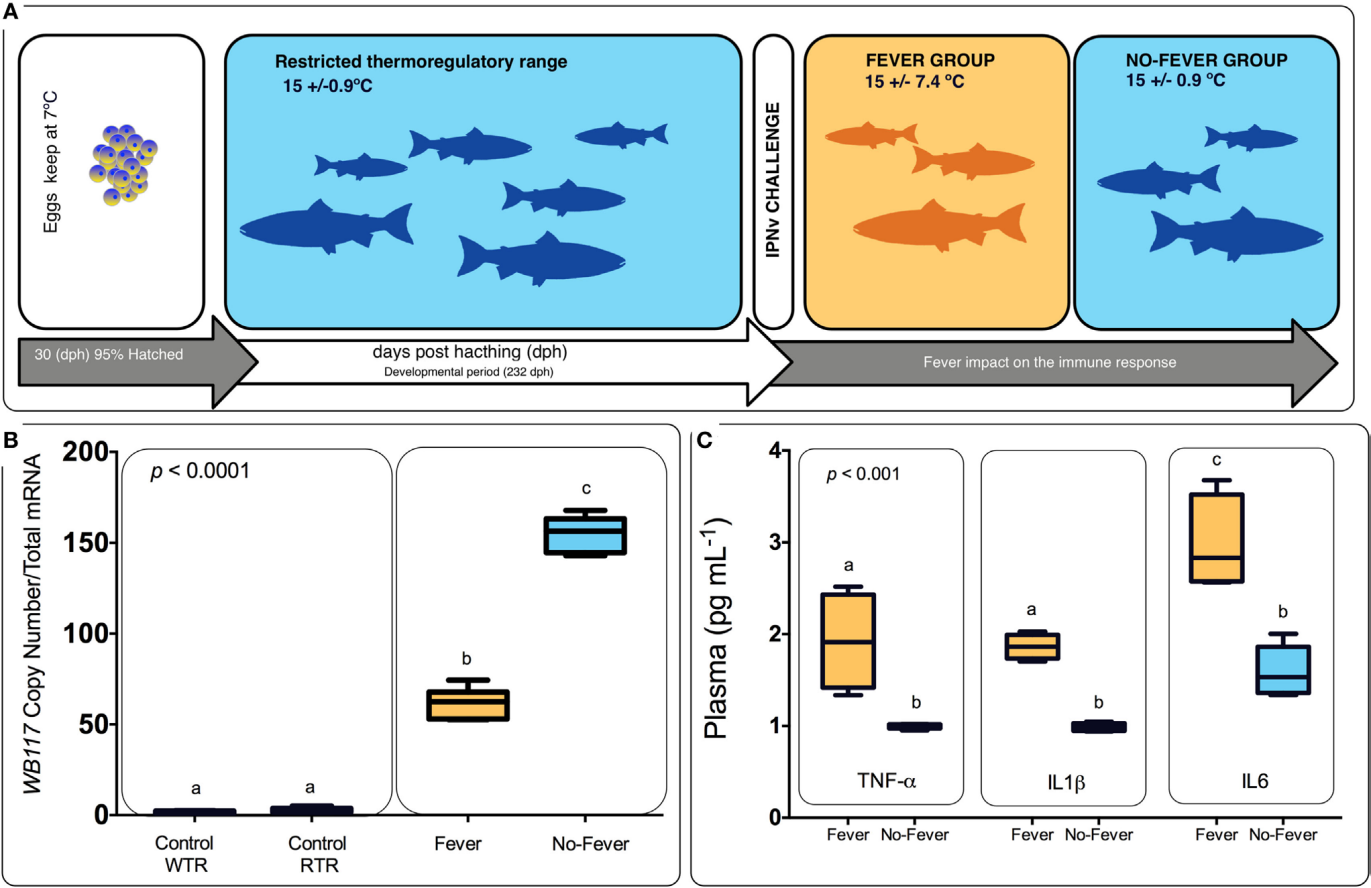
Experimental design. **(A)** Experimental design diagram showing thermal treatments after viral challenge. Temperature treatments are color-coded; blue represents the restricted (no fever group Δ_T_ 0.9°C) and orange represents the wide range (fever group, Δ_T_ 7.4°C). Experimental groups were (i) virus infected with infectious pancreatic necrosis virus (IPNv) (10 × 10^5^ PFU/mL^−1^) by immersion under constant normothermic (preferred temperature) conditions (no fever), (ii) virus infected with IPNv (10 × 10^5^ PFU/mL^−1^) by immersion in a temperature gradient (fever), (iii) control with no gradient (control RTR), and (iv) control in a temperature gradient (control WTR). **(B)** IPNV viral load during behavioral fever. Mean and SE of IPNV copy number per nanogram total RNA for the WP2 IPN viral segment. Box and whiskers plots registered differences in mRNA abundances between control and viral challenged individuals. **(C)** Indirect ELISA detection of proinflammatory cytokine release on the plasma in response to IPNv challenge. Box and whiskers plots registered differences between the unlike thermal group. Significance symbols correspond to the *p*-values for two-way ANOVA test between the different individuals. Unlike letters denote significantly different mRNA levels between the treatment.

All animal experiments conformed to the British Home Office Regulations (Animal Scientific Procedures Act 1986), the animal research was authorized by the Universidad de Concepcion Institutional Animal Care and Use Committee. Thermal experiments were carried out at the ThermoFish Lab, Biotechnology Center, University of Concepcion, Concepción, Chile. *S. salar’s* embryos were obtained from AquaGen S.A. (Melipeuco, Chile) in December 2015. Hatchery conditions used were as described by Boltaña et al. (18). Briefly, fish embryos were maintained in a recirculating freshwater systems (temperature = 7 ± 0.7°C) with a constant photoperiod of light: dark (LD) until hatching. Then, when 95% of the embryos hatched (i.e., 30 days post-hatching), temperature was gradually incremented until reaching 15 ± 0.9°C (Figure 1). The fish were maintained for 9 months and fish were fed twice a day on a commercial diet (Biomar, S.A., Puerto Montt, Chile).

### IPNv Challenge and Mortality Rates

Following the fish were challenged with IPNv. In brief, *in vivo* infection of salmon with IPNv was performed by immersion using dechlorinated water from stock tanks following protocols previously described (37). Clarified supernatant from IPNv-infected CHSE-214 cell monolayers (10 × 10^5^ PFU/mL^−1^) was added to 5 L water tanks containing the fish (*n* = 60). After 120 min, fish were separated in two groups and placed in the two-different experimental thermal set up: (a) constant temperature (mean temperature 15 ± 0.9°C, no fever) and (b) temperature gradient (mean temperature 15 ± 7.4°C, fever). In parallel, 60 fishes in another tank were treated by adding 100 mL virus free cell culture supernatant to the water (mock infected), separated into two groups and placed in two-different conditions; (c) constant temperature (mean temperature 15 ± 0.9°C, control RTR) and (d) temperature gradient (mean temperature 15 ± 7.4°C, control WTR). All temperatures were recorded each day at the same time of the day. Experimental groups were (i) virus infected with IPNv (10 × 10^5^ PFU/mL^−1^) by immersion under constant normothermic (preferred temperature) conditions (no fever), (ii) virus infected with IPNv (10 × 10^5^ PFU/mL^−1^) by immersion in a temperature gradient (fever), (iii) control with no gradient (control RTR), and (iv) control in a temperature gradient (control WTR). Three independent replicates by experimental group were carried out (*n* = 10 by replicate). Three fish by replicate (*n* = 9) were anesthetized by MSS2 and the pronephros and blood were sampled 24 h post-infection. Blood samples were individually collected from the caudal vein and centrifuged. Supernatant sera were kept frozen at −20°C. To achieve the mortality analysis, the remaining fish (*n* = 7 by replicate) were keep in the behavioral tanks and scored daily for a 10-day period; we recorded abdominal distension, exophthalmia, impaired swimming, and skin/fin base hemorrhages. To verify that IPNv was the cause of death, liver and kidney and Gills of moribund or dead fish were fixed in 10% buffered formalin for histopathological and immunohistochemical examination (data not shown). In all groups, behavioral data were recorded as described following.

### Behavioral Studies

The experimental thermal gradient was carried out in 2.5 m^3^ tanks (105 cm × 15 cm × 15 cm) divided with five transparent Plexiglas screens to create six equal interconnected chambers. Each screen had a hole at the center (3 cm diameter; 10 cm from the bottom) to allow fish to move freely between chambers. Three video cameras provided continuous monitoring of each tank chamber. During the experiment, temperatures were recorded for 10 s every 15 min throughout 24 h (96 recorded events). Four groups of fish (*n* = 10 for each group) were introduced into chamber 4 in the evening (6:00 p.m.) and filming began at 6:00 a.m. the next day, providing a 12-h acclimation period. The distribution of fish into the six compartments was monitored over time with video cameras and the number of fish in each compartment was counted manually from the images captured at each successive 15 min, resulting in 96 measurements per day. Thermal gradients were achieved with a mean difference in temperature of 13.564°C between chambers 1 and 6 by simultaneously heating chamber 6 (mean temperature = 20.725 ± 0.712°C) and cooling chamber 1 (mean temperature = 7.161 ± 0.476°C). All temperatures were recorded each day at the same time of the day. The mean number of fish observed per day in each compartment + SD (*n* = 30) was registered for each experimental group (no fever, fever, control RTR, and control WTR).

### IPNv Recovery (qPCR)

Pronephros samples were used to inspect the virus load after 24 h post-infection. Total RNA used for high-throughput transcriptome sequencing, RT-qPCR and flow cytometry analysis, was previously analyzed in order to attest and confirmed the successful completion of infection with (IPNv). To do so, an RT-PCR was performed for IPNv load estimation by targeting the virus segment VP2 region using primer WB117 and a Universal ProbeLibrary probes as previously described (38–40).

### Pronephros RNA Extraction

Nine individuals from each challenge (WTR control, control mock, fever, and no fever) were selected for RNA extraction using 30 mg of tissue after 24 h post-infection. Total RNA was individually isolated using Ribo-Pure™ Kit (Ambion^®^, USA) according to the manufacturer’s instructions. RNA concentration and purity were estimated using the NanoDrop 1000 Spectrophotometer (Thermo Scientific, MA, USA), while the RNA integrity number (RIN) was evaluated through the 2200 TapeStation (Agilent technologies, CA, USA) using the R6K screen tape. Samples with RIN ≥8 and 260/280 ratio ≥1.8 were used for library construction.

### High-Throughput Transcriptome Sequencing: Library Construction and Illumina Sequencing

Total RNA from pronephros of each condition group (no fever, fever, control RTR, and control WTR) were pooled, considering three randomly selected individuals per replicate (*n* = 9 individuals by treatment). From these pools, six barcoded libraries were constructed using the KAPA Stranded mRNA-Seq Kit (KapaBiosystems, MA, USA) according to the manufacturer’s instruction. Briefly, from 3 µg of pooled total RNA, mRNA was isolated and fragmented, followed by double-stranded cDNA synthesis. Later, ends were repaired and adenylated at the 30 end in order to perform the NEXTflex^®^ RNA-Seq Barcodes (BiooScientific, TX, USA) ligation and final PCR amplification. Library validation was based on length distribution as estimated with the 2200 TapeStation (Agilent Technologies, CA, USA) using D1K screen tape and reagents (Agilent Technologies, CA, USA). Libraries with mean length peaks above 250 bp were used for sequencing and were quantified by qPCR using the Library Quantification Kit Illumina/Universal (KapaBiosystems, MA, USA) according to the manufacturer’s instructions. Two biological replicates were used for each condition, and sequencing was performed with the Miseq (Illumina) platform using a run of 2 × 250 paired-end reads at the Laboratory of Biotechnology and Aquatic Genomics, Interdisciplinary Center for Aquaculture Research (INCAR), Universidad de Concepción, Chile. The *de novo* assembly sequence data are available from corresponding author on request.

### Gene Ontology (GO-DAVID Analysis) and Interactome Analysis

Enrichment of specific gene ontology (GO) terms among the set of probes that are specific to challenges was assessed to correlate a specific set of mRNAs within a pronephros. In all GO analyses, Ensembl gene identifiers were tested using DAVID Bioinformatics Resources^1^ (41, 42). Enrichment of each GO term was evaluated through use of the Fisher’s exact test and corrected for multiple testing with FDR [pFDR < 0.05 (43)]. We applied a Bonferroni correction to account for multiple tests performed. Each gene set comprised of at least four transcripts that shared the same GO biological process or annotation term. The final GO immune-enrichment analysis was carried out with the Cytoscape 3.5.1.^2^ Topological analysis of individual and combined networks was performed with Network Analyzer, and jActiveModules 2.2 was used to analyze network characteristics (44, 45). GO analyses were conducted with the Biological Network Gene Ontology (ClueGO, version 2.0) plugin (46) used for statistical evaluation of groups of proteins with respect to the current annotations available at the Gene Ontology Consortium.^3^ In addition, we conducted a complementary analysis with ClusterMaker cytoscape plugin (47), using the MCL algorithm to search protein–protein interaction network modules derived from tandem affinity purification/mass spectrometry (TAP/MAS). This approach clustered the network into modules based on PE score to indicate the strength of the node association and given a fixed set of genes with high protein–protein affinity (interactome cluster nodes).

### Chromatin Extraction

Three individuals from each challenge group (no fever, fever, control RTR, and control WTR) were selected for chromatin extraction using Chromatin Extraction Kit (Abcam, Cambridge, United Kingdom) according to the manufacturer’s instructions. For each individual, 10 mg of pronephros (12 h post-infection) was cut into small pieces and transferred to Dounce homogenizer. Then 50 µL of extracted chromatin was transferred to microTUBE AFA, and fragmented into 100–300 pb fragments using the following program: duty cycle = 10%, cycles per burst = 200, temperature (bath) = 4°C, cycle time = 90 s ON and 10 s OFF, cycles = 16. The size of sheared chromatin was verified on agarose gel 1% electrophoresis (120 min, 60 V) before starting the immunoprecipitation step. Length of sheared chromatin obtained was between 100 and 300 bp with peak size of 200 pb. Chromatin solution was stored at −80°C until its use.

### Chromatin Immunoprecipitation (ChIP)

Immunoprecipitation was performed using ChIP Magnetic One Step (Abcam, Cambridge, United Kingdom) following the manufacturer’s instructions. Chromatin solution was pooled and 10 μg per reaction was used. Samples were incubated with four different antibody Chip grade were used: anti-H3K27me3 (ab6002, Abcam), anti-H3K4me (ab8580, Abcam), anti-H3K4me1 (ab8895, Abcam), H3K27ac (ab4729, Abcam) (0.8 μg/well) during 120 min in a rolling shaker. After several washings, reversal of cross-links was carried out. This step consisted in two incubations with Proteinase K (0.025 µg/µL), (at 60°C for 15 min and then 95°C per 5 min) followed by the release and elution of DNA using magnetic stand. Purified DNA was quantified using Nanodrop ND-1000 (Thermo Fisher Scientific, MA, USA), stored at −20°C and then sent to Omega Bioservices for Illumina High Throughput sequencing.

### ChIP-Sequencing and Bioinformatics Analysis

Chromatin immunoprecipitation was carried out by the customer and the eluted DNA fragments were subjected to library prep using KAPA Hyper Prep Kit (KAPABIOSYSTEMS). Briefly, end-repair, A-tailing, adapter ligation, and PCR reactions were performed following the manufacturer’s recommended protocols. Thermal cycling conditions used were 98°C for 45 s followed by optimal cycles of 98°C for 15 s, 60°C for 30 s, and 72°C for 30 s, then 72°C for 1 min and hold at 4°C. PCR cycles were determined according to the sample starting amount. The post-ligation cleanup and post-amplification cleanup were performed with Mag-Bind RxnPure Plus magnetic beads (Omega Bio-tek, Norcross, GA, USA) to remove short fragments such as adapter dimers. The libraries were qualified and quantified using Agilent 2200 Tapestation instrument (Agilent Technologies, Santa Clara, CA, USA). The samples were then pooled in equimolar concentrations and sequenced in 2 × 150 bp paired-end read setting on Illumina HiSeq machine (Illumina, San Diego, CA, USA). For the bioinformatics analysis, first the fastq reads were trimmed into 50 bp using customized perl script. Paired-end reads were pooled together and treated as single-end reads to enhance mapability. Follow, the trimmed reads were then aligned to Salmon genome ICSASG_v2 using Bowtie2 (2.3.0). Mapping statistics can be found in Alignment.xlsx. The ChIP-seq peak call was performed by Homer (v4.8). The histone peak calling function was used with default parameters. Corresponding control samples were used as background for the peak calling. Each identified peak must has its peak height >1 reads per million or 10 reads per ten million and >4-fold higher than the input. Finally, ChIP-seq peaks were mapped to their nearest genes by Homer. For each histone modification marker, a pool of identified peaks was constructed by merging peaks from all samples (Bedtools 2.26.0). The average ChIP-seq tag density was calculated using Homer and plotted in R (3.3.2). Heatmap for ChIP-seq tag density was generated using seqMINER (1.2).

### Flow Cytometry Analysis

Salmon pronephros cells were isolated and processed for flow cytometry analysis (pronephros of each condition WTR control, control mock, fever, and no fever). Cell isolation was performed by disaggregating tissue through Falcon^®^ cell strainers (100 µm) with DMEM media plus 10% FBS and 1% glutamax. The cells were pelleted by centrifugation at 1,200 rpm for 10 min. Maisey et al. (48) methodology was carried out with some modifications. In brief, pronephros cell was re-suspended and blocked with PBS plus 2% FBS immunofluorescence (IF) media. Cells were incubate for 1 h at 4°C with primary antibody anti-CD4-1 (protein G- or affinity-purified Abs) (49). Then, cells were suspended again with IF media containing secondary antibody BV421 Goat Anti-Rabbit IgG Clone Polyclonal (565014, BD Bioscience, NJ, USA) and Alexa Fluor^®^ 488 Goat Anti-Mouse IgG H&L (ab150113, Abcam, Cambridge, United Kingdom) and incubated 1 h at 4°C. Finally, cells were washed and suspended once again in 200 mL IF media prior to analysis. For auto fluorescence measurement, cells were suspended with IF containing no Ab, whereas for isotype controls, cells were treated only with the corresponding conjugated secondary Ab. A BD LSRFortessa™ X-20v flow cytometry was used to analyze sample and at least 10,000 events were recorded for each sample. Recorded events analyzed using the FlowJo software.

### Absolute RT-PCR Validation in Pronephros

RT-qPCR was performed using the StepOnePlus™ Real-Time PCR System (Applied Biosystems, Life Technologies, NC, USA), and each assay was run in triplicate using the Maxima SYBR Green qPCR Master Mix (2×) (Bio-Rad, NC, USA). cDNA used in qPCR assays was first diluted with nuclease free water (Qiagen, Hilden, Germany). Each qPCR mixture contained the SYBR Green Master Mix, 2 µL cDNA, 500 nmol/L each primer, and RNase free water to a final volume of 10 µL. Amplification was performed in triplicate on 96-well plates with the following thermal cycling conditions: initial activation for 10 min at 95°C, followed by 40 cycles of 15 s at 95°C, 30 s at 60°C, and 30 s at 72°C. A dilution series made from known concentrations of plasmid containing the PCR inserts was used to calculate absolute copy numbers for each of the genes examined. Previously published primers were used (Table S1 in Supplementary Material).

### ELISA Measurement of Plasma Cytokine *TNF-α, IL-1β*, and *IL-6*

Blood plasma was obtained from individual salmon (*n* = 9 individuals treatment; no fever, fever, control RTR, and control WTR) after 12 h post virus infection and stored at −80°C until use. To determine the detection of *IL-6, TNF-α*, and *IL-1β* in plasma samples of *S. salar*, indirect ELISA was performed according Morales-Lange et al. (50). Briefly, each plasma sample was diluted in carbonate buffer (60 mM NaHCO_3_, pH 9.6), planted (in duplicated for each marker) at 35 ng/µL (100 µL) in a Maxisorp plate (Nunc, Thermo Fisher Scientific, Waltham, MA, USA) and incubated overnight at 4°C. After, each well was blocked with 1% bovine serum albumin (BSA) for 2 h at 37°C. Then, plates were incubated for 90 min at 37°C with the primary antibody anti-synthetic epitope (diluted in BSA) of TNF-α (diluted 1:500), *IL-6* (diluted 1:500), and *IL-1β* (diluted 1:500). Later, the second antibody-HRP (Thermo Fisher Scientific, Waltham, MA, USA) was incubated for 60 min at 37°C in 1:7,000 dilution. Finally, 100 µL per well of chromagen substrate 3,3′,5,5′-tetramethylbenzidine single solution (Invitrogen, CA, USA) was added and incubated for 30 min at room temperature. Reaction was stopped with 50 µL of 1N sulfuric acid and read at 450 nm on a VERSAmax microplate reader. Primary antibodies against cytokines were produced according Bethke et al. (51). Synthetic epitope peptides were used for immunization in CF-1 mouse for *IL-6* and *TNF-α* (52) and New Zealand rabbit for *IL-1β* (53). For validation, antibody efficiency was determined by the calibration curve of the antibody against the synthetic peptide used for the immunization through indirect ELISA (54) and antibody specificity was verified by Western blot as described before in Schmitt et al. (53).

## Results

### Behavioral Fever and the Impact of Thermal Choice Upon the Transcriptome

**Figure 2:**
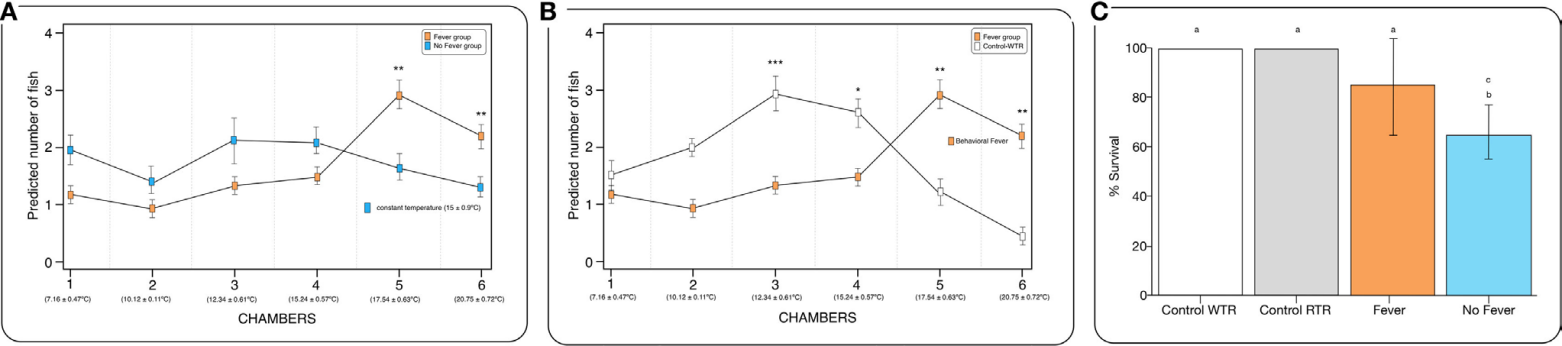
Behavioral fever in infectious pancreatic necrosis virus (IPNv)-challenged *Salmo salar*. **(A)** Frequency of chamber occupation in individual fish challenged with IPNv (fever vs no fever). The blue box represents fish challenged with IPNv under constant temperature. The orange box represents fish challenged with IPNv under thermal gradient tank (*n* = 10 mean ± SD, *p, 0.05; **p, 0.01; ***p, 0.001). **(B)** Frequency of chamber occupation in individual fish challenged with IPNv in a thermal gradient tank (orange boxes) and control individuals (no infected group in thermal gradient tank, white boxes control-WTR; *n* = 10 mean ± SD, *p, 0.05; **p, 0.01; ***p, 0.001). All individuals used were conditioned at constant temperature under 9-month (15 ± 0.9°C). **(C)** Survival percentage, the results are presented as the percentage of surviving fish after 10 days post viral challenge (mean ± SD, two-way ANOVA *F*_2,39_ = 14.89; unlike letters denote significantly differences).

**Figure 3:**
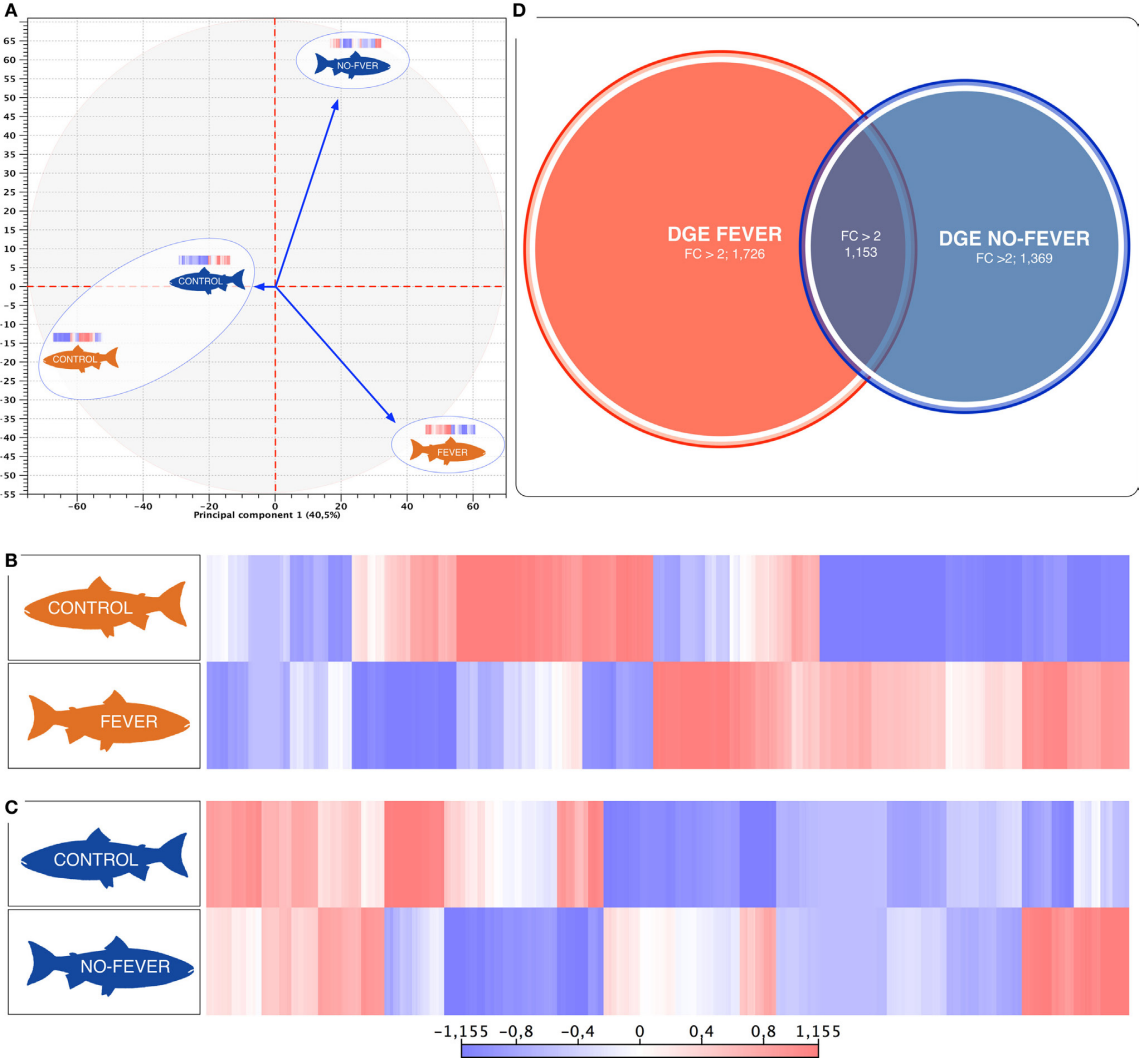
Parallel effects of behavioral fever and temperature on the gene expression levels. **(A)** Principal component 1 explains 40.5% of overall variance in gene expression and is correlated with the temperature effect on the gene expression mainly on the behavioral fever group (*p* = 0.04, *R*^2^ = 0.10, *n* = 24). **(B)** Heatmaps of log2-transformed gene expression levels for rank-associated genes of thermal wide range group (fever). **(C)** Heatmaps of log2-transformed gene expression levels for rank-associated genes of thermal restricted range group (no fever). Values are shown after controlling for differences in means among social groups; 0 roughly corresponds to mean expression levels, red upregulated genes, blue downregulated genes. **(D)** Venn diagram on the distribution of mRNAs across the unlike thermal group. The overlapping expression of identified RNA transcripts from thermal group are depicted in different colors: fever (red), no fever (blue).

The behavioral analysis modeled the number of fish that were found in chambers with higher temperatures (chambers 5 and 6) as a function of the two temperature groups (control WTR and control RTR), the challenge groups (fever and no fever), and their interaction (four groups in total). The Wald test highlight that in the fever group, the number of fish found in higher temperatures (chamber 5 or 6) was significantly higher in comparison to the control group (*p* < 10^−3^) (Figures 2A,B; Table S2 in Supplementary Material). The results suggest that the IPNv challenged group with access to a thermal gradient tank (fever group) preferably chose the warm chambers (*n*°= 5 and 6) and the unchallenged fish prefer chambers 3 and 4 (thermo-preferendum, Wald test *p* < 10^−3^). The kinetics of the behavioral fever show that in the fever group fish perform behavioral fever throughout the day, however, the mostly fish develop fever past 12 h of infection (Table S2 in Supplementary Material). The transcriptomic analysis was evaluated using RNA-seq of salmon challenged with the birnavirus IPNv under two different thermal conditions: (1) wide thermal range (fever group) and (2) restricted thermal range (no fever). Each group was composed of nine individuals (three individuals by each replicate), resulting in one thermal profile for each group (see Figures 3A–C). Viral load was quantified using RT-qPCR assays for the genome segment WP2 of the IPNv (39, 40, 55). Viral loads were higher in individuals that have not developed behavioral fever, in contrast to the fever group (Figure 1B). Differences in the response to IPNv infection in the fever and no fever groups were significant both in transcript number (total number of differentially expressed transcript over the control, one-way ANOVA *p* < 0.01) and intensity (fold change FC > 2) (Figures 3B,C). After filtering for contigs that mapped exclusively to the *S. salar* genome, we considered the normalized expression levels of 4,248 transcripts that were differentially expressed in all group. We identified a specific transcriptomic signature for each group in the RNA-seq data (with 1,726 and 1,369 in fever and no fever group, respectively). Principal component analysis highlighted a significant correlation of gene expression data with each fish group (*R*^2^ = 0.10, *p* < 0.03; Figure 3A). Following on we further analyzed the effect of behavioral fever on the variation in mRNA abundance for each specific transcript identified of interest.

We used a linear mixed-effects model in which residual variation in gene expression levels, after taking into account differences across the fish groups, was treated as the response variable. The temperature condition (fever and no fever) was incorporated as a fixed effect, and the significance of the thermal conditions on the gene expression was assessed based on the strength of this effect. We identified an association between inter-group gene expression variation and fever group for 2,879 genes (67.7% of the 4,248 genes we considered; false discovery rate = 10%; Figure 3D). Within the set of 2,879 fever associated genes, 1,726 genes (49.6% of all genes we considered) were more specifically expressed in the fever group (Figures 3B,D), and 1,369 genes (32.2%) were specifically expressed in individuals housed at a restricted thermal range (no fever group, Figures 3B,C). The results highlight a juxtaposed transcriptomic response between wide and restricted thermal range during development and after the viral infection highlighting the effects of thermal choice and behavioral fever upon underlying molecular processes.

**Figure 4:**
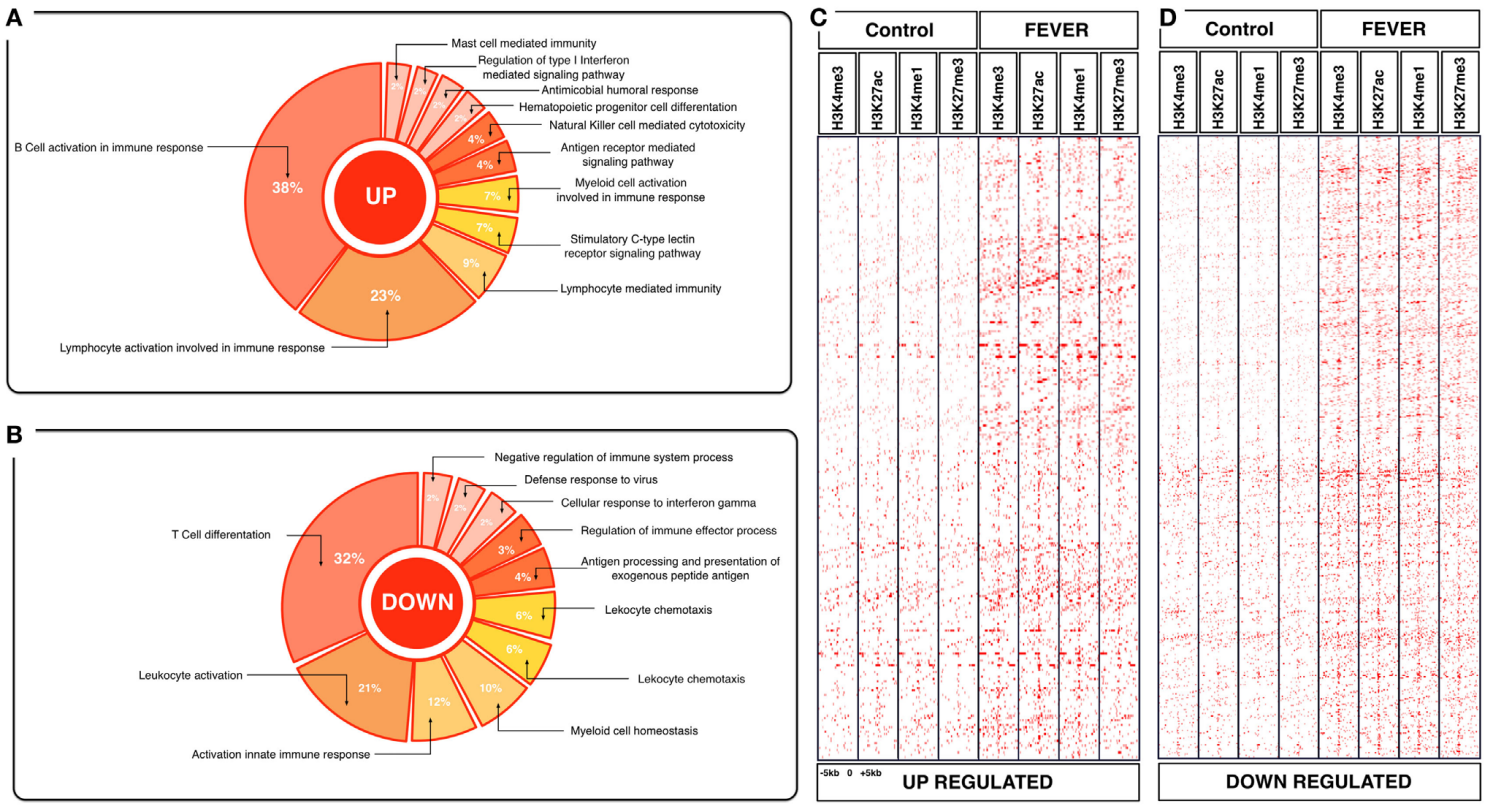
Effects of behavioral fever on the immunological process and histone landscape. Gene ontology enrichment analysis of transcripts involved in immune process in pronephros of fever individuals (GO DAVID and ClueGo Cytoscpae Plugin). **(A)** Upregulated genes and **(B)** downregulated genes. **(C,D)** Heat map showing chromatin immunoprecipitation-seq read density (log2 transformed) for the indicated of 1,726 WTR^+/+^ genes referenced in salmon ENSEMBL database (down and up) at equivalent genomic regions in 5-kb windows upstream and downstream of the TSS binding sites (TSS; assumed to be at the 5 kp end of annotated genes). The specific genes were sorted into categories based on enrichment H3K4me1, H3K4me3, 3K27me3, and H3K27ac.

**Figure 5:**
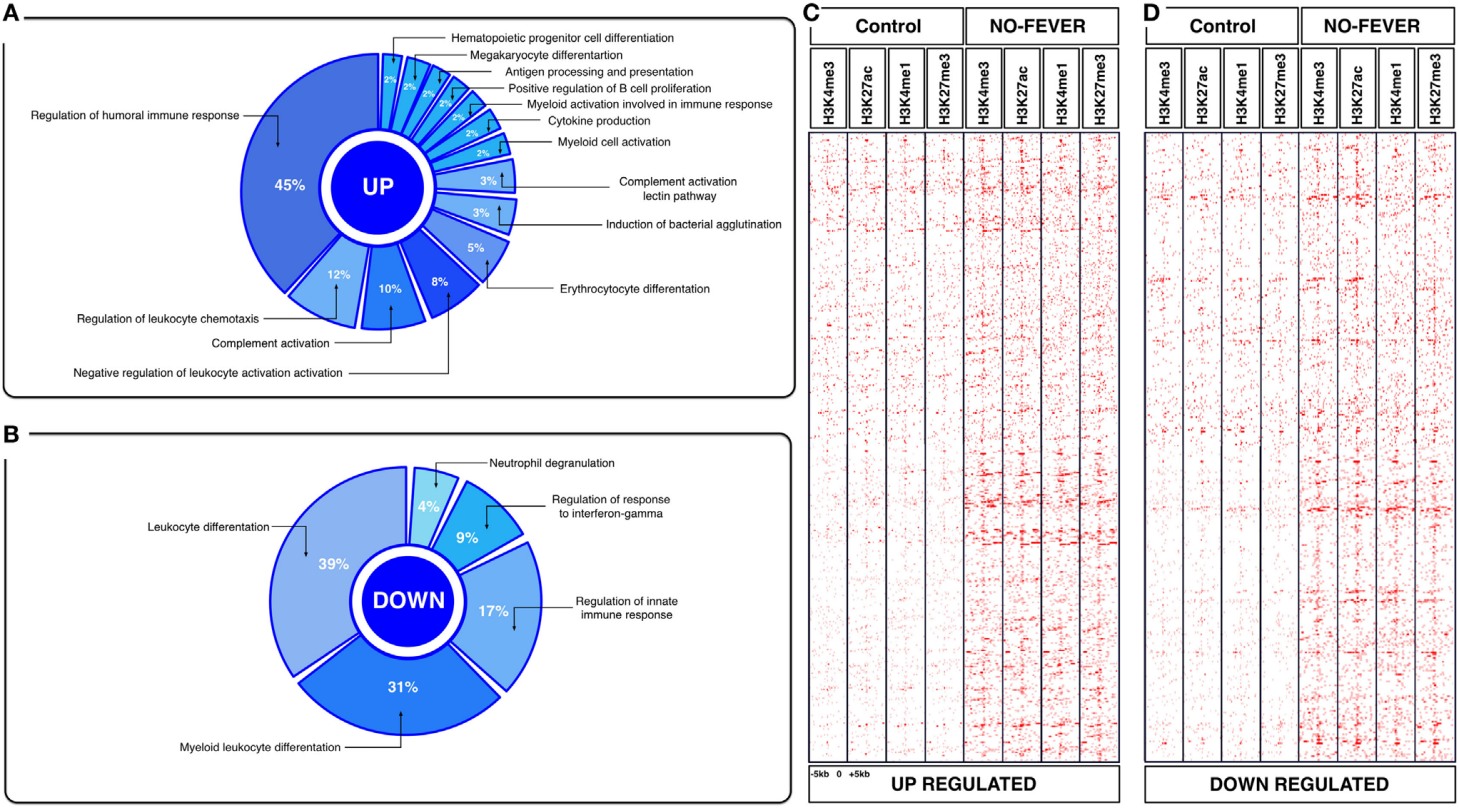
Effects of constant temperature on the immunological process and histone landscape. Gene ontology enrichment analysis of transcripts involved in immune process in pronephros of no fever individuals (GO DAVID and ClueGo Cytoscpae Plugin). **(A)** Upregulated genes and **(B)** downregulated genes. **(C,D)** Heat map showing chromatin immunoprecipitation-seq read density (log2 transformed) for the indicated of 1,369 RTR^−/−^ genes referenced in salmon ENSEMBL database (down and up) at equivalent genomic regions in 5-kb windows upstream and downstream of the TSS binding sites (TSS; assumed to be at the 5 kp end of annotated genes). The specific genes were sorted into categories based on enrichment H3K4me1, H3K4me3, 3K27me3, and H3K27ac.

**Figure 6:**
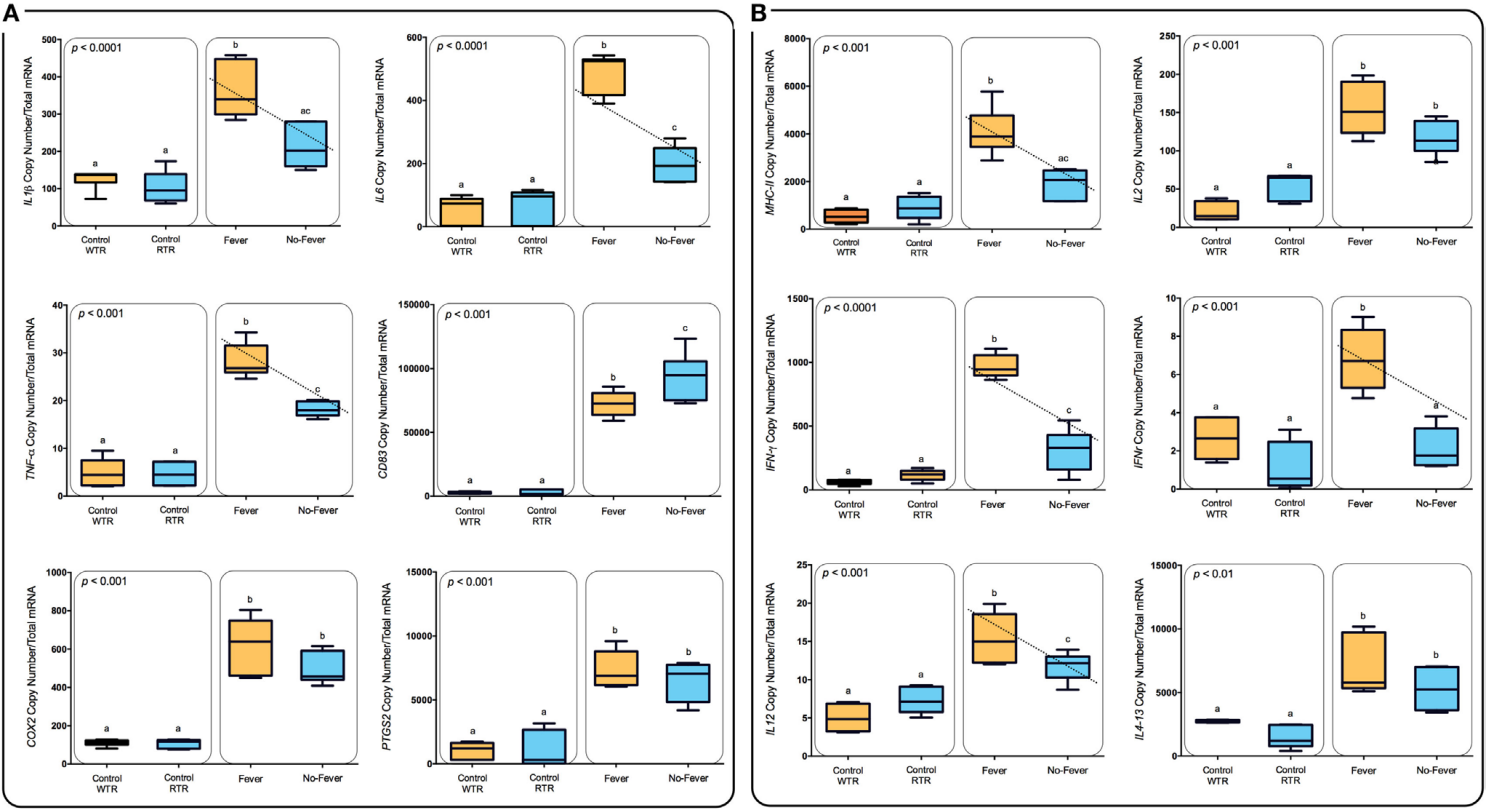
Expression profiles among lymphocyte-related immune genes. **(A)** Box and whiskers plots registered differences in mRNA abundances (back-transformed, ordinary least-squares means) between the thermal treatments are shown for each gene (*MHC-II, IL-2, IFN-γ, IFNR-γ, IL-12, and Il4-13*). Unlike letters denote significantly different mRNA levels between the treatment (two-way ANOVA). **(B)** Expression profiles among inflammation-related immune genes. Box and whiskers plots registered differences in mRNA abundances (back-transformed, ordinary least-squares means) between the thermal treatments are shown for each gene (*IL1β, IL-6, TNF-α, CD83, COX-2, and mPGES-1*). Unlike letters denote significantly different mRNA levels between the treatment (two-way ANOVA).

To explore the biological functions associated with these genes, we incorporated a protein–protein interaction network derived from TAP/MAS ClusterMaker Cystoscope-plugin (47). We selected the modules with over representation of node–node interactions (immune enrichment analysis) that were found in each thermoregulatory range and conducted GO functional analysis with GO-DAVID (42) and the ClueGO Cytoscape plugin (46). GO-DAVID and ClueGO identified a significant enrichment of functional GO categories related to the immune response (Figures 4A,B and 5A,B). We identified a strong signal for enrichment of specific immune response categories in the fever group associated gene set as a whole. Here, we observed enrichment for interferon signaling (*p* = 0.01, *q* = 0.05), B-cell activation (*p* = 0.01, *q* = 0.05), and lymphocyte activation involved in immune response (*p* < 0.02; proportion of false discoveries at this *p* value threshold, *q* = 0.30) among genes highly expressed in the behavioral fever group (Figures 4A,B) suggesting a strong induction of innate and adaptive immune responses (Figure 4A). Examples of such genes (Figures 6A,B) include *IFNR-γ* and *IFN-γ* (a receptor for the proinflammatory cytokine INF, which is associated with neutrophil migration into injured tissue), *IL4/13, IL-2, IL1-2* (which is associated with the transcriptional response to T-cell stimulation) and proinflammatory cytokines, which include *mPGES-1* (proinflammatory signaling molecule that is positively regulated during the fever process) and *IL1, IL-6, TNF-α, COX-2* (Figure 6A) and also by cytokine release (Figure 1C). The ClueGo analysis reflected a coherent immunological function and fever group, specifically we found that the largest module (38% of the genes; mean *r* = 0.58) was enriched for B cell activation in the immune response, followed by specific immune-related gene subsets including lymphocyte activation involved in immune function (*p* = 0.002, *q* = 0.08), response to *IFN-γ* (*p* = 7.50 × 10^−6^, *q* = 9.0 × 10^−5^) and myeloid cell activation involved in immune response (*p* = 6.31 × 10^−4^, *q* = 3.80 × 10^−3^). The present results highlight that behavioral fever promote the activation of specific immunological/adaptive processes further supports the observation that behavioral fever induces a coordinated functional response at the level of the transcriptome that is juxtaposed of the registered at individuals kept at constant temperature (no fever group, Figures 5A,B). Once we established a potential mechanism whereby behavioral fever potentiates the accumulation of response-specific mRNAs in the responding tissue, we sought to test the contribution of fever to survival. After 10 days post virus challenge, the behavioral fever group (fever individuals, Δ_T_ 6.4°C) showed no clinical signs of infection and low mortality rates in significant contrast to those held under constant conditions (Figure 2C).

### Behavioral Fever Influences Lymphocyte Activation

**Figure 7:**
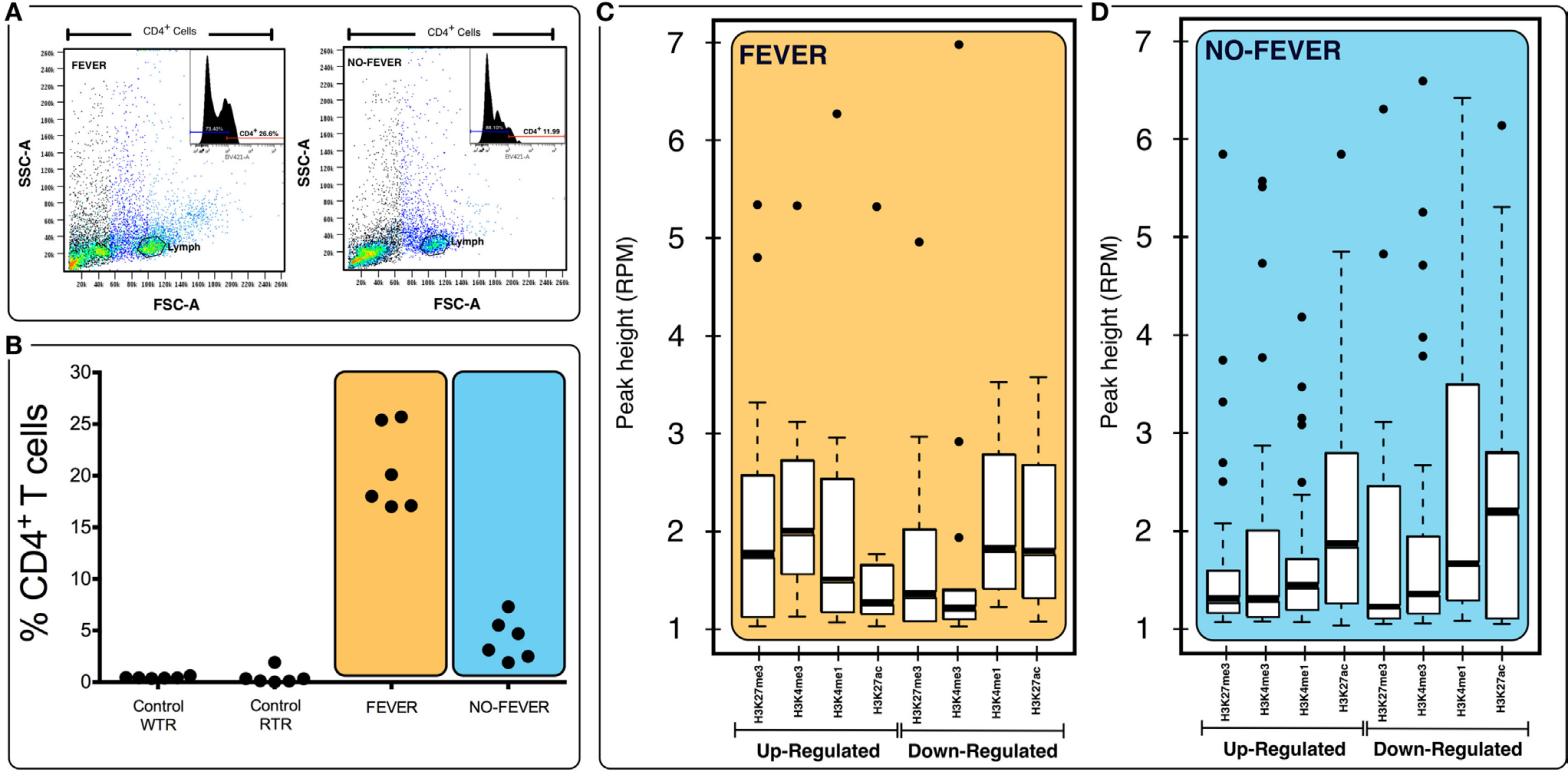
Effects of tissue composition mediated regulation on fever behavior and temperature gene expression levels. **(A)** Behavioral fever individuals exhibit higher proportions of CD4^+^ T cells in pronephros tissue (*p* = 0.031, *n* = 60; *y*-axis shows the percentage of T-cell proportions after infectious pancreatic necrosis virus challenge). Panel **(B)** shows the lymphocyte population and the inset show an example data for histogram of fever and no fever individuals; *x* axis shows staining for CD4^+^ (helper) T cells (% of cells positive for CD4-BV421 antibody). **(C,D)** Box plots for chromatin immunoprecipitation-seq RPM values of fever and no fever group of genes. Box plots of adjusted (adj.) input normalized H3K4me3 reads per kilobase per million mapped reads (RPKM), **(C)** values at TSS (fever genes, *n* = 1,726) **(D)** and TSS-containing domains (no fever genes, *n* = 1,369). Box and whiskers correspond to the highest and lowest points within the 1.5× interquartile range.

We used fluorescence-activated flow cytometry (FACS) analysis to estimate the proportion of the two-main cell populations (CD4^+^ T cells) in pronephros samples from 60 fish. The no fever group individuals had a reduced proportion of CD4^+^ T cells (*p* < 0.05, *n* = 10; Figures 7A,B), whereas the group expressing behavioral fever had a significantly increased, twice, the proportion of CD4^+^ T cells in contrast to the no fever group during the viral infection (*p* < 0.05, *n* = 10). Interestingly, the CD4^+^ population was also significantly higher in the fever group in comparison to the non-infected group (both control group WTR and RTR, respectively, *p* < 0.05, *n* = 10). The results emphasizes the failure of the individuals that have not developed fever to increase the CD4^+^ T cell population in contrast to the observed in the fever group individuals (*p* < 0.05, *n* = 10; Figures 7A,B).

### Regulatory Mechanisms Underlying the Thermoregulation and Fever Behavior

Previous results and those obtained in this study reinforce our understanding of how both thermal choice and the expression of behavioral fever during infection can strongly impact the regulation of gene expression. Furthermore, in this study, we have also shown the impact of temperature and the fever response upon the CD4^+^ T lymphocyte populations in salmon. Next, we investigated whether differences in gene expression observed could be influenced by genome histone modification during viral infection. To explore this hypothesis, we examined changes in histone modifications (ChIP-seq; HiSeq-Illumina 10×) from pronephros DNA. We then investigated the relationships between histone methylation and the recruitment of markers for active transcription (H3K4me3 and H3K27ac), for repressed transcription (H3K27me3 and H3K4me1), and gene expression.

To assess whether histone methylation levels might contribute to the observed gene expression associations, we checked histone methylation data in the 1,726 and 1,369 genes previously selected as specific gene sets for fever and no fever individuals. We found that active transcription driven by methylation data from H3K27ac, H3K4me3 clearly distinguished between both thermal individuals (Figures 7C,D; Figures S1A,B in Supplementary Material). The histone methylation data distinguished upregulated and downregulated genes from specific groups of genes from each experimental group (Figures 4C,D and 5C,D). Specifically, we observed epigenetic features of transcriptomic gene sets triggered exclusively by individuals expressing behavioral fever that were associated with a specific methylation pattern (Figure 7C; Figure S1A in Supplementary Material). In fever group, also was observed an increase of the H3K4me3 a histone hallmark frequently linked with transcription initiation providing a “window of opportunity” for the enhancer activation. By contrast, individuals reared under a restricted thermoregulatory (no fever group, Figure 7D; Figure S1B in Supplementary Material) H3K4me3 modification was weakly detectable. In his thermal group, the enrichment of H3K27me3 also was slightly increased (Figure 7D; Figure S1B in Supplementary Material).

In individuals expressing behavioral fever, H3K4me3 and H3K27ac histone modifications were highly noticeable in several GO where active transcription of genes related to the immune adaptive response occurs (Figures 4C,D and 7C). Unlike H3K27ac, the repressive transcriptional marker H3K27me3 did not appear to have a specific pattern for these GO processes in fever group of individuals (Figures 4C,D and 7C). In contrast to the pattern observed for the behavioral fever individuals, not-fever group shows an increase in repressed transcription activity linked to H3K27me3 modification across several biological processes related to the previous enrichment analysis for immune response (Figure 5D). In the individuals that have not developed fever (no fever group) H3K4me1 modification was also evident. Thus, the observed gene expression patterns appear to be tightly related to differential histone methylation data that is significantly different under the different thermal regimes investigated. Epigenetic regulation therefore may explain, at least in part, how thermal choice and the expression of behavioral fever under infection can potentiate the immune response in ectotherms.

## Discussion

The fact that the “*fever*” response to infection and injury has been maintained throughout at least 600 million years of evolution strongly suggests a positive benefit to immunity and overall survival (24, 25, 27). Our results highlight the influence of thermal choice and the importance of behavioral fever to mount a successful immune response during infectious episodes. Furthermore, our data opens a significant new avenue supporting the increasingly recognized link between increasing temperature and epigenetic regulation in fish (56, 57). Critically in this and our previous studies, we report that the impact of temperature is not observed across the entire transcriptome but focused into specific profiles. These transcriptome profiles correlate to functional differences measured in lymphocyte proliferation and cytokine release suggesting different immune response modules are influenced in a specific manner. The significant variation observed in immune response in ectotherms might partly be explained by unsuitable environmental settings resulting in non-optimal gene-temperature interactions (18). In our current design, individuals move freely throughout the thermal gradient (wide thermal range) after a pathogen challenge. As other abiotic variables (e.g., salinity or pH) may also play a role modulating response to the virus (58, 59), additional experiments should be needed to robustly test this hypothesis.

In fish, few studies of epigenetic modification have been associated with environmental effects during the development (60–63), and to our knowledge, no studies have associated the impact of epigenetic modification on the immune response. Here, we show that observed differences in the transcriptome (5,602 mRNAs) and associated GO processes in response to viral infection between the fever and no fever groups could be explained at least in part by epigenetic modification. The histone modifications observed between the different groups were relatively low when compared with homeothermic species or in the context of human diseases such as cancer (64–66). However, the observed changes in epigenetic marks driven by behavioral fever are higher in comparison to other studies addressing the influence of environmental factors upon other fish species (67–69). By testing epigenetic modification in our “thermal choice” model and linking this to behavioral fever-associated gene expression, we have identified a regulatory role for the epigenome in fish. This regulatory step appears to be directly coupled to environmental temperature and impacts upon immune performance including cellular activity and the humoral response. In ectotherms, it is hypothesized that coupling of the immune response to fever promotes the survival (5, 70). However, currently there are not studies that explain the mechanism through which the immune response is increasing during the fever response. Our results show for the first time, evidence that the adaptive value of the fever response lie at the level of epigenetic–environment interaction affecting systemic and specific immune response.

Remarkably, we identified a strong and significant increase in CD4^+^ T cells in the pronephros of infected salmon expressing behavioral fever. Although the presence of a definitive Th1 response in fish is currently under debate our data supports the development of a Th1 response. In parallel to increased CD4^+^ T cells, *IL-6, Il-1β, IL-12, TNF-α, IFNR-γ*, and *IFN-γ* mRNAs are all significantly induced in IPNv infected salmon supporting the development of a specific Th1 response in salmon. Importantly these mRNAs are all significantly higher in IPNv-infected fish expressing behavioral fever. In mammals, the sensory vanilloid receptor 1 (TRPV1) has been shown that in addition to a thermal sensing role can also regulate the signaling and activation of CD4^+^ T cells and cytokine release (71, 72). Current studies in salmon suggest a key role for TRP receptors in regulating the behavioral fever response in fish (73). *IL-4* is critical to the development of Th2 responses in mammals and in the fish *IL-4 like mRNAs* have been described and named *IL4/13* (74, 75). Interestingly, *IL4-13* mRNA was upregulated in the pronephros as a result of IPNv infection, however, there were no differences between fish in the WTR and RTR environments. A similar expression pattern was observed for *IL-2* which supports differentiation and maintenance of both Th1 and Th2 states (76). Thus, it appears that behavioral fever at least within the time scales measured strongly favors the development of a Th1 type response to viral infection. Further studies will address CD8^+^ T cells due to their significance during a viral infection (71, 72). Humoral response components, *COX2* and *PTGS2* mRNAs, representing the prostaglandin system were upregulated, however, no “fever” effect was evident. In previous studies we have shown significant increases in PGE_2_ plasma concentrations (5) suggesting that behavioral fever is more likely to affect enzymatic activity by increased temperature rather than at the mRNA level.

The present results show that fish expressing behavioral fever, challenged with virus increase H3K4me3 on genes encoding factors involved in innate immunity and lymphocyte differentiation. The pathogenic stimulation of human monocytes also increases H3K4me3 in a group of innate immune genes (31). By contrast, before viral infection, increased amounts of H3K27me3 and reduced basal expression of innate target genes are sustained for individuals that have not or cannot develop behavioral fever. A similar pattern of histone influence on gene expression related to immune response has been observed in murine macrophages challenged with endotoxin that were previously exposed to β glucans (77, 78) which be a mechanistic link regulate LPS tolerance. It is known that epigenetic alterations lead to the priming of genes encoding host defense molecules that respond specifically to pathogens (79, 80). This systemic protective mechanism, through epigenetic modifications, likely has significant adaptive value as remodeled cells would respond more robustly to pathogens. Thus providing the individual with a selective immunological advantage (29, 81). Our findings document the role of epigenetic regulation on the immunological pathways activated in salmon expressing behavioral fever during viremia. These dynamic epigenetic elements and the observed thermal-coupling effect during fever clearly influences specific defense modules such as the Th1 response during viral infection and may play a critical role in the development of trained immune responses in fish. Our analysis highlights the key functions of the epigenetic modifications in regulating the immune response during the fever process and is likely where the behavioral modification leading to warmth-seeking initiates. We detected high viral load in pronephros 24 h post-challenge in both thermal treatment (fever and no fever individuals), additionally, we were able to recognize significant differences in the accumulative mortality. Early detection of IPNv after immersion challenge has been documented on rainbow trout (81). The observed mortality in the “no fever” group are in concordance with previous studies of fish challenged at a constant temperature, which suggests that high tropism of IPNv observed in pronephros is followed by an increase of the mortality after 7 days of virus infection (82–84). Although the beneficial or deleterious effects of fever are still debated (7–10), the present results extend our previous observations that behavioral fever in ectotherms has a positive adaptive value due to increased survival (5, 17).

In conclusion, our results highlight the close interaction between behavioral fever, immune performance particularly the development of a Th1-like response and significant epigenetic regulation at the molecular level in a model of viral infection in the *Atlantic salmon*, a species of considerable economic interest for aquaculture. Our study highlights the importance of creating environments where experimental animals are able to express a “normal” behavior therefore uncovering important underlying regulatory circuits. We propose that behavioral fever acts as an integrative signal that promotes specific epigenetic modifications that drives a protein production in responding lymphocyte populations. This, in turn, leads to increased efficacy of immune defense traits and provides a positive adaptive value to the host. Our data demonstrate the extensive plasticity of the immune response in fish and therefore by extension provides insight into other ectothermic organisms. This approach delineates thermal thresholds, in this case for *Atlantic salmon*, where the potential costs of adverse thermal environments are traded for improved immunological responses and therefore increased survival. The observed temperature epigenetic synergy leading to increased survival has implications toward understanding the molecular basis of disease resistance in ectotherms, and provides opportunity to understand the evolutionary consequences of behavioral fever in ectotherms.

## Ethics Statement

All animal experiments conformed to the British Home Office Regulations (Animal Scientific Procedures Act 1986), the animal research was authorized by the Universidad de Concepcion Institutional Animal Care and Use Committee.

## Author Contributions

The study was conceived by SB with important input from SM. AA and NS performed the experiments. SB analyzed the data. SB, SM, and NS performed the model simulations and provided extensive additional input. SB: funding acquisition. SB and SM drafted the manuscript with substantial contributions from all other authors.

## Conflict of Interest Statement

The authors declare that the research was conducted in the absence of any commercial or financial relationships that could be construed as a potential conflict of interest. The handling Editor declared a past co-authorship with the authors SB and SM.

## Footnotes

^1^http://david.abcc.ncifcrf.gov/tools.jsp (Accessed: December 10, 2017).

^2^http://www.systemsbiology.org (Accessed: December 10, 2017).

^3^http://www.geneontology.org (Accessed: December 10, 2017).

## Supplementary Material

Figure S1 Average H3K4me1, H3K4me3, 3K27me3, H3K27ac RPKM values of 5′ and 3′ sides, and flanking regions of for **(A)** fever genes TSS-containing domains (*n* = 1,726), and **(B)** no fever genes TSS-containing domains (*n* = 1,369) broader than 10 kb: infectious pancreatic necrosis virus challenged (red), control (blue).

Table S1 mRNA primer sequences used for absolute RT-qPCR analysis.

Table S2 Kinetic of the behavioral fever. The table shows the frequency of chamber occupation in individual fish challenged with infectious pancreatic necrosis virus during 24 h post-infection, the video cameras provided continuous monitoring of each tank chamber. The frequency was recorded for 10 s every 15 min throughout 24 h.
