# Supplementary figures and images for: Behavioral Fever Drives Epigenetic Modulation of the Immune Response in Fish
Source: Front Immunol. 2018 Jun 4;9:1241. doi: 10.3389/fimmu.2018.01241 (PMC5994863; doi:10.3389/fimmu.2018.01241)

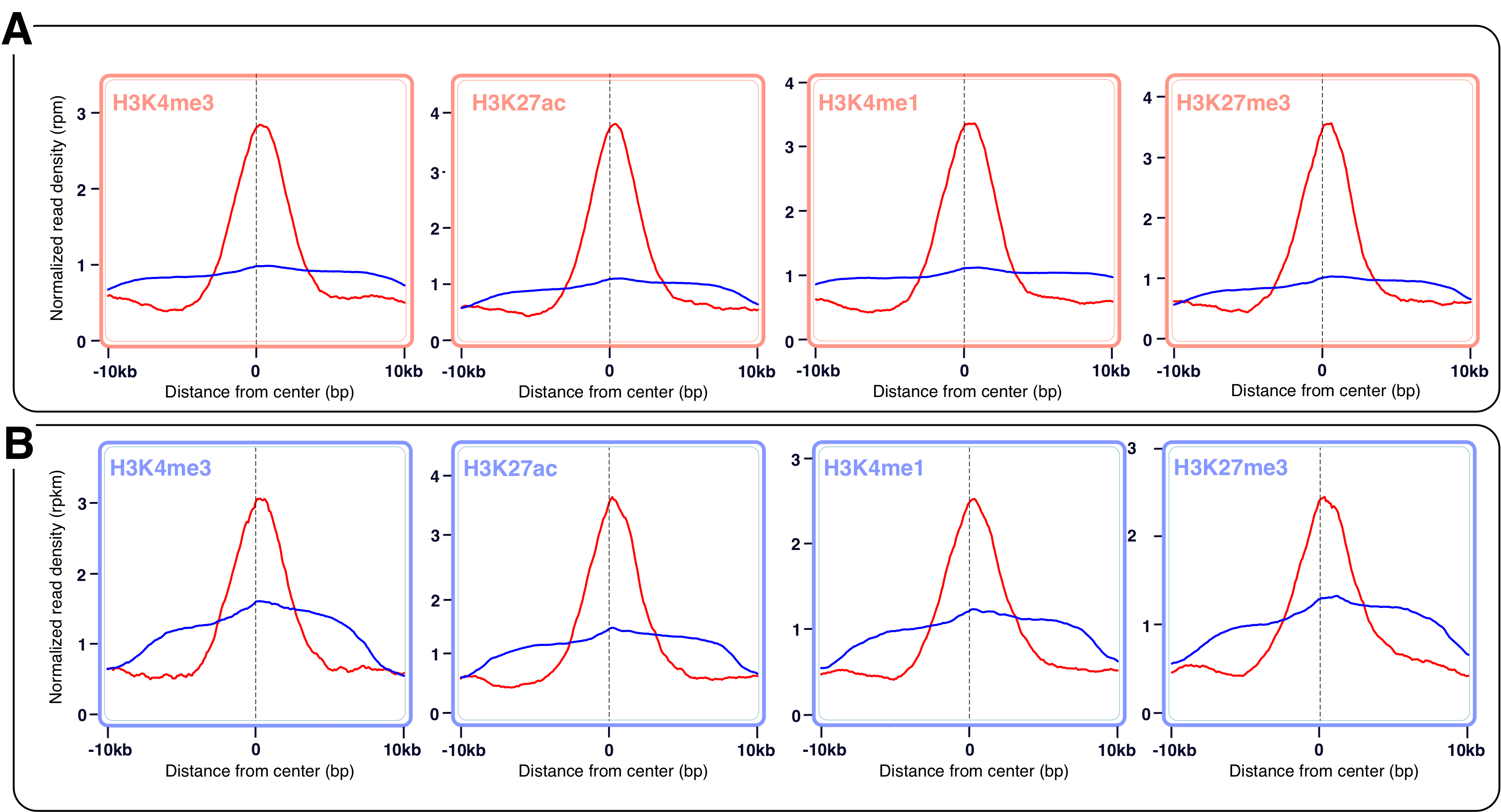

Supplement: Figure S1 — Average H3K4me1, H3K4me3, 3K27me3, H3K27ac RPKM values of 5′ and 3′ sides, and flanking regions of for (A) fever genes TSS-containing domains (n = 1,726), and (B) no fever genes TSS-containing domains (n = 1,369) broader than 10 kb: infectious pancreatic necrosis virus challenged (red), control (blue). [file image_1.tiff]
