# Supplementary material for: Behavioral Fever Drives Epigenetic Modulation of the Immune Response in Fish
Source: Front Immunol. 2018 Jun 4;9:1241. doi: 10.3389/fimmu.2018.01241 (PMC5994863; doi:10.3389/fimmu.2018.01241)
Supplement: Table S1 — mRNA primer sequences used for absolute RT-qPCR analysis. [file table_1.docx]

| **Name** | **Gen** | **Primer 5'-3'** | **Tamaño (bp)** | **E**  **(%)** | **Tm**  **(°C)** | **GenBank accesión n°** |
| --- | --- | --- | --- | --- | --- | --- |
| TNF-α | Tumor Necrosis Factor-Alpha | F: AGGTTGGCTATGGAGGCTGT | 250 | 108 | 62 | NM_001123589 |
|  |  | R: TCTGCTTCAATGTATGGTGGG |  |  |  | NM_001123590 |
| IL1β | Interleukin 1 Beta | F: CGTCACATTGCCAACCTCAT | 200 | 98 | 60 | AY617117 |
|  |  | R: ACTGTGATGTACTGCTGAAC |  |  |  |  |
| IL-6 | Interleukin 6 | F: CAGCGGAGAGGTAGTCTGGA | 139 | 117 | 60 | DQ866150 |
|  |  | R: CGTCCAGAAAGCAGTGGACT |  |  |  |  |
| PGD2 | Prostaglandin D2 synthase | F: GCTGTCCAGTATGATGACTTTGC | 195 | 95 | 58 | AF281353.1 |
|  |  | R: GGCACATTCACCGTTCTTGG |  |  |  |  |
| COX-2 | Cytochrome C Oxidase II | F: CAGTGCTCCCAGATGCCAAG | 99 | 94 | 60 | AY848944.1 |
|  |  | R: GCGAAGAAGGCGAACATGAG |  |  |  |  |
| IFNγ | Interferon Gamma | F: CTAAAGAAGGACAACCGCAG | 159 | 107 | 60 | AJ841811.1 |
|  |  | R: CACCGTTAGAGGGAGAAATG |  |  |  |  |
| WB117 | Infectious pancreatic necrosis virus, protein VP2 | F: GCGGTTCGACTTCATTCTACA | 100 | 116 | 60 | U48225.1 |
|  |  | R: GAGCTTGTCACGGAGACCAC |  |  |  |  |
| MHCIIβ | Major histocompatibility complex, class II, beta | F:AGAAGCCTGGAACAAAGGTCCTGA | 109 | 95 | 60 | AJ438974.1 |
|  |  | R: AACTGTCTTGTCCAGTATGGCGCT |  |  |  |  |
| IL2 | Interleukin 2 | F: GTTGCAGCATTGGCCTGTTCC | 110 | 99 | 60 | FN356744.1 |
|  |  | R: AGTCCTGTTGATGTGGTGTTGC |  |  |  |  |
| IFNγ-r | Interferon Gamma Receptor 1 | F: AAGCATGGGGATTCTGGTCCT | 161 | 103 | 60 | NM_001124416.1 |
|  |  | R: CCGATATGTGACAGTATGGAAGC |  |  |  |  |
| IL12 | Interleukin 12 | F: TCAGTAAGCTGAGGGTCCGC | 110 | 92 | 60 | HG917954 |
|  |  | R: TCCTCCTTTTTGCCTCCCGT |  |  |  |  |
| IL4-13 | Interleukin-4/13A | F: GACCACCACAAAATGCAAGGA | 146 | 92 | 60 | AB574339 |
|  |  | R: GGTTGTCTTGGCTCTTCAC |  |  |  |  |
| CD83 | CD83 Antigen | F: GCACCTGTAGGAGAGCAGAACC | 89 | 80 | 60 | DQ339141.1 |
|  |  | R: TCCCTTTCTTCTGATTGGTCTGT |  |  |  |  |

**Supplementary Table 1**.- Primers used for qRT-PCR.
